# Fast track hip and knee arthroplasty: impact of different hospital care levels

**DOI:** 10.1515/med-2026-1391

**Published:** 2026-03-18

**Authors:** Martin Betz, Jürgen Konradi, Felix Wunderlich, Roman Paul, Michael Clarius, Manfred Krieger, Philipp Drees, Ulrich Betz

**Affiliations:** Department of Orthopaedics and Trauma Surgery, University Medical Centre of the Johannes Gutenberg University, Mainz, Germany; Institute of Physical Therapy, Prevention and Rehabilitation, University Medical Centre of the Johannes Gutenberg University, Mainz, Germany; Institute of Medical Biostatistics, Epidemiology and Informatics, University Medical Center of the Johannes Gutenberg University, Mainz, Germany; Vulpius Hospital GmbH, Bad Rappenau, Germany; Department of Orthopedic Surgery, GFR Hospital Rüsselsheim, Rüsselsheim, Germany

**Keywords:** endoprosthesis, treatment process, optimization

## Abstract

**Objectives:**

Total knee and hip replacements are among the most common surgical procedures. Optimization of the treatment process is of great relevance and requires verification of effectiveness in different settings. The PROMISE study was carried out to achieve this.

**Methods:**

An optimized treatment process was implemented in 3 German hospitals with different levels of care. A total of 1,887 patients were included. The WOMAC Score was established as the outcome parameter at 3, 6 and 12 months after surgery. A mixed model for repeated measurements was used to estimate site-specific effects as well as the limits of the corresponding confidence intervals. To demonstrate homogeneous results across all sites, these outcomes needed to fall within a range defined by the overall effect ± the minimally clinically important difference (MCID), using a 95 % confidence interval.

**Results:**

All site-specific results ranged from (points [CI-limits]) −29.2 [−29.7; −28.7] to −24.4 [−24.5; −24.3] and were therefore within the range defined by the overall treatment effect and the MCID (25.4 ± 10).

**Conclusions:**

We could demonstrate homogeneous site-specific effects of the optimized process in the three most different settings of the German healthcare system. Therefore, an essential prerequisite for a system-wide rollout has been met.

## Introduction

Total knee and hip replacements are among the most common surgical procedures with over 187.000 primary total hip arthroplasties (THA) and more than 155.000 primary total knee arthroplasties (TKA) registered in the German endoprosthesis register for 2024 [1]. Given the high volume of these procedures, optimizing the treatment process is of significant relevance, necessitating a thorough evaluation of effectiveness across various settings. The application of enhanced recovery after surgery (ERAS) guidelines [2] for an optimized treatment process (OTP) was initially developed for colorectal cancer surgeries. The protocol was subsequently adapted for use in elective knee and hip arthroplasty [3] and has shown significant advantages over traditional perioperative processes [4]. These benefits include significantly shortened LOS, reduced transfusion rate and lower 30-day postoperative mortality without increasing postoperative complications or readmission rate [5], offering considerable improvements for both patients and the healthcare system. In recent years, studies in various European countries, including Scotland [6], Denmark [7], and Spain [3], have been conducted to evaluate the adoption and effectiveness of perioperative processes based on ERAS criteria. This has shown that OTPs can be successfully realized in different healthcare systems. However, there is still a knowledge gap regarding whether the success of an OTP is independent of a specific site. To verify this, a shared standardized OTP had to be tested in as most different settings as possible. The multicenter PROMISE study [8] (process optimization by interdisciplinary and cross-sectoral care using the example of patients with hip and knee prostheses) was initiated to address these needs. The feasibility of implementing the protocol across all the included centers has already been demonstrated [9]. The analysis presented here examines the results to determine whether they are homogeneous across the study centers, which represent different levels of care. Beside feasibility, this is a second crucial prerequisite for a possible system-wide rollout of the OTP.

## Materials and methods

### Treatment

We implemented a standardized OTP [8] in three hospitals of different care levels (a regional hospital, an orthopedic clinic, and a university hospital) in different regions of Germany (Hesse, Baden-Württemberg and Rhineland-Palatinate). The process corresponds in most aspects to the recommendations of the ERAS Society, which, in a 2019 consensus statement, published standardized guidelines for the perioperative management of primary total knee and hip arthroplasty [10]. Only the recommendations regarding preoperative smoking and alcohol cessation could not be implemented, as recruitment for the PROMISE study had already begun by the time they were published.

The protocol included the following: As usual, the indication for surgical intervention is determined based on the criteria set in the respective guidelines [11], 12]. The indication is followed by a preoperative risk assessment, which consists of blood tests to detect anemia and thrombosis, as well as various questionnaires to assess the patient’s health and social situation. During a one-day briefing, patients received comprehensive preoperative information from the respective professional groups about the surgical procedure, the various types of anesthesia, mobilization on the day of surgery, and multimodal pain management.

With the exception of patients with existing contraindications, there is a fixed medication regimen consisting of intraoperative intravenous administration of corticosteroids (to regulate the surgical stress response for PONV prophylaxis) as well as the use of local infiltration analgesia with adrenaline (for pain management and to reduce the risk of postoperative bleeding during early mobilization) and intravenous and local injection of tranexamic acid (to further reduce the risk of bleeding). Both spinal and general anesthesia are permitted, the insertion of drains and the use of a tourniquet are avoided where possible. In the postoperative course, the use of opioids is limited as far as possible and by day 8 the sole remaining pain medication, if possible, is etoricoxib, which we recommend in all cases for the prevention of heterotropic ossification. In addition to a dry wound and the ability to perform activities of daily living, the discharge criteria included functional aspects such as walking using crutches for at least 150 m and being able to walk up one flight of stairs. Once the discharge criteria were met, patients were discharged home and were to begin an outpatient or inpatient rehabilitation program no later than two weeks after surgery, if possible with a PROMISE partner.

Inclusion criteria: The patient suffers from osteoarthritis, meets the standardized criteria for elective THA or TKA, and is able to understand the nature and extent of the study.

Exclusion criteria: Life expectancy of less than one year (e.g., advanced cancer), any condition that might preclude elective surgical intervention, or medical or psychological factors that would prevent the patient from participating or providing written informed consent.

Data collection and statistical analysis: The transformed Western Ontario and McMaster Universities Osteoarthritis Index (WOMAC 0 (best result) − 100) was established as the outcome parameter preoperative and at 3, 6, and 12 months after surgery. In order to evaluate whether comparable results have been achieved, a homogeneity approach as proposed by Ring et al. [13] was used. A mixed model for repeated measurements (MMRM) was set up to model the change in WOMAC over the observation period of 12 months. This allowed the inclusion of all visits for each patients and is somewhat robust to missing values. We included sex, age, ASA at baseline, and BMI as confounders. The sites were included using weighted coding, with weights proportional to the number of included subject at the respective site as proposed by Ring et al. The subjects were included as random factors in order to take the repeated measurements into account and to allow for correct estimation of standard errors. Additionally, a first-order autoregressive correlation (AR(1)) structure was assumed, as it is plausible that patients with positive outcomes in one visit will experience further beneficial results in the following visit. Using estimated covariance matrix of the model and the delta method [14], it was possible to estimate site-specific effects as well as corresponding confidence intervals. The limits of the obtained confidence intervals were compared to the effect that was achieved overall in PROMISE. The two-sided 90 %-confidence limits needed to be included within a range of the overall effect ± MCID (10 points) [15] to demonstrate a homogenous result in all sites using a 5 % significance level. This approach which is also common for equivalence testing, is a two one-sided test (TOST) [16], each single limit of the 90 %-confidence intervals represents a one-sided test with significance level of 5 % to exclude the MCID-margins. Thus, all confidence limits need to fulfill this condition simultaneously therefore two-sided 90 %-confidence intervals suffice to achieve a 5 % one-sided significance level per comparison. Analyses were performed using R 4.4.3 [17]. The trial was approved by the ethics committees of Rhineland-Palatinate [837.533.17 (11,367)], Baden-Wuerttemberg [B-F-2018-042], and Hesse [MC 84/2018]. Written informed consent was obtained from all participants.

## Results

### Demographics

A total of 1887 patients were included in the study, with 530 treated at the university hospital, 916 at an orthopedic specialized hospital, and 441 at a regional hospital. Analysis of the demographic data displayed in Table 1 shows that the mean BMI values were similar across all three hospital types. At the university hospital and the regional hospital, more female than male patients underwent surgery, whereas the orthopedic specialized hospital had an almost equal distribution of male and female patients. In addition, a higher proportion of patients with a preoperative ASA score >2 were treated at the university hospital compared to the other two hospitals. For the distribution of hip/knee patients and surgeries on one or both joints in the various groups, see Table 1.

**Table 1:** Comparison of demographic data (age, sex, BMI, and ASA score) for the different locations.

	University hospital	Orthopedic specialized hospital	Regional hospital
Age ,years (mean, SD)	67.4 (11.0)	65.2 (9.0)	68.5 (10.5)
Female (n, percentage)	306 (57.7 %)	466 (50.9 %)	278 (63.0 %)
Male (n, percentage)	224 (42.3 %)	450 (49.1 %)	163 (37.0 %)
BMI ,kg/m^2^ (mean, SD)	29.2 (6.3)	29.2 (5.4)	29.4 (5.7)
ASA score 1 (n, percentage)	19 (3.7 %)	86 (9.8 %)	17 (4.1 %)
ASA score 2 (n, percentage)	249 (47.9 %)	584 (66.4 %)	297 (72.4 %)
ASA score 3 (n, percentage)	242 (46.5 %)	209 (23.8 %)	96 (23.4 %)
ASA score 4 (n, percentage)	10 (1.9 %)	1 (0.1 %)	0 (0 %)

### Western Ontario and McMaster Universities Osteoarthritis Index

The results for the WOMAC-scores were different at all measurement points in the respective study centers. The cohort from the university hospital had the highest values (worst result) before and after surgery. However, with an average of 40.2 points, this group achieved the greatest improvement (for all results see Table 2).

**Table 2:** Results of the WOMAC score at the three sites over one year.

	University hospital	Orthopedic specialized hospital	Regional hospital
WOMAC preop (mean, missing)	58.1 (29)	42.6 (19)	43.4 (9)
WOMAC 3 month (mean, missing)	25.5 (113)	20.0 (184)	22.7 (126)
WOMAC 6 month (mean, missing)	22.3 (118)	15.4 (207)	17.6 (130)
WOMAC 12 month (mean, missing)	17.9 (152)	11.5 (233)	13.6 (148)

### Treatment effects

The overall treatment effect within the promise study using the MMRM was a change in WOMAC score of −25.4 [−31.5; −19.4]. Site-specific treatment effects ranged from −29.2 [−29.7; −28.7] to −24.4 [−24.5; −24.3] (Table 3). The estimates of the MMRM can be found in Supplementary Table S2. The site-specific effects are adjusted for the confounders sex, age, ASA at baseline, and BMI. If the type and number of endoprostheses implanted are also included in the model as confounders, the results do not contradict the results presented here (see Table 3).

**Table 3:** Site-specific effects in the three PROMSE sites and the corresponding confidence limits.

Site	Effect	Lower 95 %-CI limit	Upper 95 %-CI limit
University hospital	−29.2	−29.7	−28.7
Orthopedic specialized hospital	−24.4	−24.5	−24.3
Regional hospital	−27.8	−28.6	−27.1

### Homogeneity analysis

**Figure 1:**
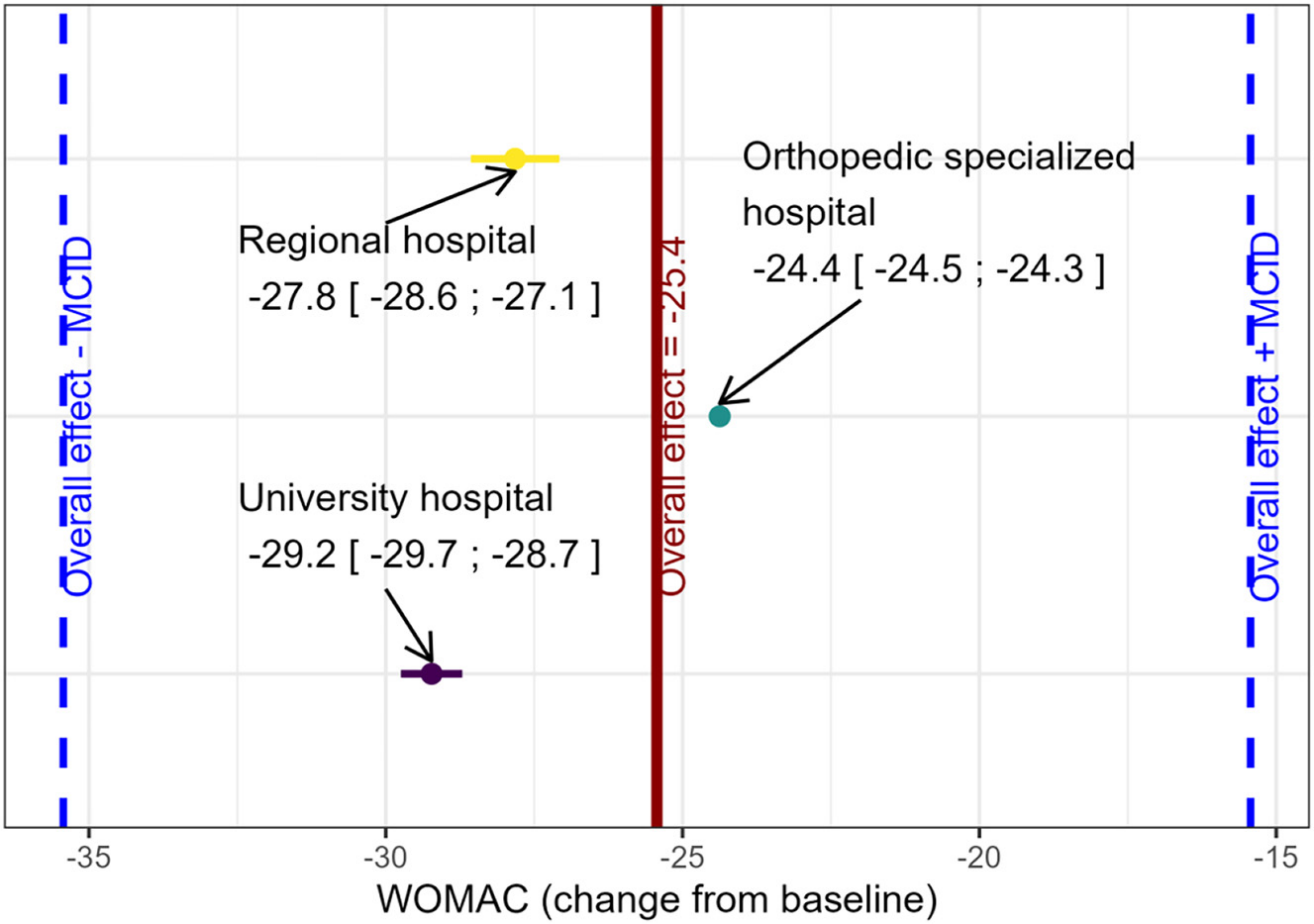
Homogeneity of site-specific treatment effects compared to the overall treatment effect observed in the PROMISE study.

All site-specific results are completely enclosed by the range defined by the overall treatment effect and the minimally clinically relevant effect (25.4 ± 10). Thus, the obtained site-specific effects can be considered homogenous compared to the overall effect (see Figure 1). In fact, the site-specific effects deviate at most by 4.3 points from the overall effect (17 % deviation). Furthermore, the maximum deviation between the site-specific effects is 5.4 points as observed between sites 1 and 2. This is still well below the minimally clinically relevant difference of 10 points.

## Discussion

There are numerous sources in the scientific literature that demonstrate success through the optimization of a care process in general [4], 18] and also specifically for hip and knee arthroplasty [3], 6], 7]. However, since the processes examined were not uniform, it is not possible to determine how the different settings affected the outcomes of the process optimization. Nevertheless, this knowledge is a fundamental prerequisite for preparing a rollout. A broad rollout within a healthcare system only appears reasonable if the optimized process proves successful regardless of the specific setting. In order to fill the existing knowledge gap, it was therefore necessary to conduct a multicenter study that implements and evaluates a standardized OTP in most different settings. The authors are not aware of any literature on this important issue. Therefore, the results of this analysis fill a very relevant gap of knowledge.

In this study, the composition of cohorts varied across the structurally different centers, as did their respective results. However, the improvements in WOMAC scores exceeded the MCID by a wide margin in all centers and are comparable to results reported in the literature [19]. It appears plausible that the university hospital – the center with the highest level of care – treated the most severely affected and overall sicker patients. Interestingly, this patient group achieved the greatest improvement, but was still unable to match the outcomes of the other groups after 12 months, even though the primary cause of complaints had been eliminated equally for all patients by the implantation of the endoprosthesis.

However, all site-specific effects are well within the range defined to show the homogeneity of the results for all cohorts. As the study centers represent very different levels of care in Germany and are also located in different regions, it can be concluded that the PROMISE care process is consistently effective regardless of the specific setting and is therefore suitable for a broad roll-out. This opportunity is already being utilized in Germany. For example, it is possible to conclude a so-called “Quality contract” [20] between hospital and health insurance company on the basis of the optimized care process examined here. These contracts enable advantages in the billing of services provided and thereby promote the dissemination of the process in the German healthcare system. A requirement for the provision of services by the Federal Joint Committee could make the dissemination of the optimized care process mandatory, just as there are already mandatory structural features for the treatment of proximal femur fractures [21], for example.

We see the strengths of the study in a prospective, multicenter design with centers from different care levels and a common, standardized, optimized treatment process. A large number of subjects were included and followed up using an internationally recognized, diagnosis-specific and comprehensive score at several points in time up to one year after the surgery. On the basis of the repeated measurements, a statistically reliable result could be determined despite a relevant number of missing results. As the centers were very different, but all belong to the same healthcare system, the results cannot be transferred to other healthcare systems without restriction. In contrast to a comparable study [22] on process optimization in cardiac surgery, the treatment groups in this study were not controlled by standard therapy groups. Due to the uncontrolled design, the influence of OTP on the results could not be determined in this study. This was justified by the fact that the practical feasibility of two fundamentally different treatment paths in the same facility appeared questionable. It was assumed that the bias would exceed the advantages of a controlled design due to insufficient differentiation of the various treatment paths [23]. The fact that, in contrast to the ERAS recommendations, the PROMISE protocol does not prescribe preoperative smoking/alcohol cessation for all three participating centers in the same way is unlikely to have had any influence on the comparability of the results between the centers. Overall, the importance of the care process appears to decrease in the longer term after the surgery. So, in a current systematic review with 47 studies and 76,971 included patients, no advantage could be demonstrated for the OTP with regard to the WOMAC score [5].

In summary, it can be stated that the OTP developed for this study for patients undergone total hip or knee arthroplasty has led to homogeneous results in hospitals with most different levels of care in Germany. This is an important prerequisite for a broad roll-out.

## Supplementary Material

Supplementary Material
