# Supplementary material for: Fast track hip and knee arthroplasty: impact of different hospital care levels
Source: Open Med (Wars). 2026 Mar 18;21(1):20261391. doi: 10.1515/med-2026-1391 (PMC12995390; doi:10.1515/med-2026-1391)
Supplement: Supplementary file 1 — Supplementary Material [file j_med-2026-1391_suppl_001.docx]

Supplementary Materials:

Supplement table 1: Distribution of hip/knee patients and surgeries on one or both joints in the various groups.

|  | All sites | University hospital | | Orthopedic hospital | | Regional hospital | |
| --- | --- | --- | --- | --- | --- | --- | --- |
|  | n | n | (%) | N | (%) | n | (%) |
| Patients | 1887 | 530 | 28% | 916 | 49% | 441 | 23% |
| Joints | 1977 | 585 | 30% | 946 | 48% | 446 | 23% |
| **Hip cohort** |  |  |  |  |  |  |  |
| Hip patients | 954 | 216 | 23% | 466 | 49% | 272 | 29% |
| Hip joints | 982 | 229 | 23% | 477 | 49% | 276 | 28% |
| Hip – one side patients | 926 | 203 | 22% | 455 | 49% | 268 | 29% |
| Hip – both sides patients | 28 | 13 | 46% | 11 | 39% | 4 | 14% |
| **Knee cohort** |  |  |  |  |  |  |  |
| Knee patients | 934 | 315 | 34% | 450 | 48% | 169 | 18% |
| Knee joints | 995 | 356 | 36% | 469 | 47% | 170 | 17% |
| Knee – one sided patients | 873 | 274 | 31% | 431 | 49% | 168 | 19% |
| Knee – both sides patients | 61 | 41 | 67% | 19 | 31% | 1 | 2% |

Supplement table 2. Estimators obtained from the MMRM including sex, age, ASA at baseline, and BMI. K1 and K2 were stand-ins for the weighted dummy coding used to obtain the site-specific estimators.

|  | Estimate | Standard error | p-value |
| --- | --- | --- | --- |
| Intercept (Overall effect) | -25.43243 | 3.69235 | < 0.001 |
| Sex (Female vs male) | -3.04331 | 0.73950 | < 0.001 |
| Age [yrs] | 0.12496 | 0.03897 | 0.001 |
| ASA (2 vs. 1) | -1.63369 | 1.49663 | 0.275 |
| ASA (3 vs. 1) | -3.94125 | 1.62229 | 0.015 |
| ASA (4 vs. 1) | -11.62906 | 5.51865 | 0.035 |
| BMI | 0.11121 | 0.06753 | 0.100 |
| K1 | -16.61207 | 2.63498 | < 0.001 |
| K2 | 4.62972 | 1.66403 | 0.005 |

Supplement table 3: Estimators obtained from the MMRM including sex, age, ASA at baseline, BMI, affected joint and number of treated joints. K1 and K2 were stand-ins for the weighted dummy coding used to obtain the site-specific estimators.

|  | Estimate | Standard error | p-value |
| --- | --- | --- | --- |
| Intercept (Overall effect) | -14.61795 | 12.88661 | 0.257 |
| Sex (Female vs male) | -2.98304 | 0.71671 | < 0.001 |
| Age [yrs] | 0.07774 | 0.03808 | 0.041 |
| ASA (2 vs. 1) | -1.58906 | 1.45028 | 0.273 |
| ASA (3 vs. 1) | -3.34569 | 1.57342 | 0.034 |
| ASA (4 vs. 1) | -10.48186 | 5.35812 | 0.051 |
| BMI | -0.02546 | 0.06685 | 0.703 |
| K1 | -19.98204 | 2.60602 | < 0.001 |
| K2 | 4.69095 | 1.61645 | 0.004 |
| Knee prostheses (1 vs. 0) | -0.28448 | 12.38034 | 0.982 |
| Knee prostheses (2 vs. 0) | -0.14974 | 12.55908 | 0.991 |
| Hip prostheses (1 vs. 0) | -7.61644 | 12.37790 | 0.538 |
| Hip prostheses (2 vs. 0) | -10.39613 | 12.73124 | 0.414 |
